# Impact of Resistance Exercise under Hypoxia on Postexercise Hemodynamics in Healthy Young Males

**DOI:** 10.1155/2018/1456972

**Published:** 2018-07-26

**Authors:** Masahiro Horiuchi, Arisa Ni-i-nou, Mitsuhiro Miyazaki, Daisuke Ando, Katsuhiro Koyama

**Affiliations:** ^1^Division of Human Environmental Science, Mt. Fuji Research Institute, Japan; ^2^Faculty of Education and Human Sciences, University of Yamanashi, Japan; ^3^Graduate School Department of Interdisciplinary Research, University of Yamanashi, Japan

## Abstract

We investigated the effects of resistance exercise under hypoxia on postexercise hemodynamics in eight healthy young males. The subjects belonged to a track & field club (sprinters, hurdlers, and long jumpers) and engage in regular physical training (1-2 h per day, 3-5 days per week). Each participant performed eight sets of bilateral leg squats with a one-minute interval under normoxia (room air) and hypoxia (13 % FiO_2_). During a 60-minute recovery, we set normoxic condition either after normoxic or hypoxic exercise. These two experimental protocols (normoxia and hypoxia) were performed in a random order with a one-week washout period. The leg squat exercise consists of 50 % 1-RM (14 repetitions) × 5 sets and 50% 1-RM (repetitions max; 7 repetitions) × 3 sets. The resting period between each set was 1 min, and a total of 91 repetitions were performed. Blood pressure, heart rate (HR), and several biomarkers were measured pre- and postexercise. The mean arterial pressure (MAP) significantly decreased after exercise compared to the pre-exercise values under both conditions (*P* < 0.05). The MAP at 20 and 30 min of recovery in hypoxia was significantly lower than in normoxia (*P* < 0.05, respectively). The antidiuretic hormone significantly increased after 60 min of recovery in both conditions; moreover, the values in hypoxia were significantly higher than those in normoxia (*P* < 0.05). The delta changes in MAP from baseline (pre-exercise) were significantly related to changes in HR from baseline in normoxia (r = 0.560,* P* < 0.001) but not in hypoxia. These results suggest that the hypoxic condition elicits greater hypotension after resistance exercise in comparison to normoxia. Moreover, the underlying mechanisms for the attenuation of hypotension after resistance exercise may differ between normoxia and hypoxia.

## 1. Introduction

In healthy individuals, an acute single bout of physical exercise elicits reductions in arterial blood pressure for approximately 2 h when compared to the baseline (pre-exercise) levels; this phenomenon is so-called postexercise hypotension (PEH) [1–3]. In addition, recent findings suggest that the magnitude of reductions in blood pressure (BP) with acute exercise may be associated with chronic reductions in the resting BP caused by exercise training [4, 5]. These results suggest that PEH with acute exercise may be relevant for predicting the long-term effects of BP changes.

Although these studies were conducted with aerobic exercise [4, 5], several studies have observed the occurrence of PEH after acute resistance exercise [6–10]. Moreover, a recent meta-analysis revealed that a single bout of resistance exercise can lower the BP in normotensive and hypertensive populations and the use of larger muscle groups resulted in greater BP reductions after resistance exercise [11]. Resistance exercise may exert some additional advantages; for example, resistance exercise is recommended for prevention of sarcopenia, wherein age-induced muscular degeneration often leads to compromised performance in activities of daily living [12]. Moreover, it has been suggested that resistance exercise in hypoxia reportedly has beneficial effects in terms of increased muscle strength and size [13, 14]. However, it should be noted that greater PEH may frequently cause syncope after exercise [15] and higher intensity exercise induces a postexercise syncope compared to lower intensity exercise [16]. These results suggest that negative effects of PEH should also be considered, in particular, higher exercise intensity (e.g., high intensity aerobic or resistance exercise). Indeed, it was reported that the arterial blood pressure reached 320/250 mmHg during the maximal bilateral leg press [17] but the pressure subsequently fell rapidly after lifting [18]. As a result, the cerebral perfusion pressure falls below the pre-exercise levels, leading to an increased risk of syncope [18, 19]. Indeed, we reported that, after prolonged leg cycling, there was a greater reduction in the mean arterial blood pressure (MAP), and the decrease in MAP was positively related to decreases in cerebral oxygenation only in hypoxia [20]. These results suggest that resistance exercise in hypoxia may involve an increased risk of syncope even for healthy individuals, indicating that clarification of the mechanisms of PEH after resistance exercise in hypoxia is important. However, little is known about how resistance exercise in hypoxia may affect postexercise hemodynamics.

The aim of this study, therefore, was to investigate the influence of resistance exercise in hypoxia on postexercise hemodynamics in healthy young males. We hypothesized that resistance exercise in hypoxia elicits greater reductions in arterial blood pressure than in normoxia. To test this hypothesis, we used bilateral leg squats at 50% 1-RM (repetition max) with the inspiration of oxygen (FiO_2_) at ~13% based on a previous study [21]. In addition, we measured several potent vasoconstrictors (antidiuretic hormone and aldosterone) and vasodilators (human atrial natriuretic peptide and adenosine) that may influence BP regulation.

## 2. Methods

### 2.1. Participants

This study was approved by the Ethical Committee of the University of Yamanashi in Japan and was performed in accordance with the guidelines of the Declaration of Helsinki (No: 201606). Eight healthy male subjects with a mean age of 19.9 ± 0.8 years, a height of 170.0 ± 3.1 cm, and a body mass of 65.1 ± 11.3 kg (mean ± standard deviation [SD]) participated in this study. The subjects belonged to the track & field club at the University of Yamanashi (sprinters, hurdlers, and long jumpers) and engage in regular physical activity (1-2 h per day, 3-5 days per week). None of the subjects had been exposed to an altitude higher than 1500 m within the six months prior to the study. All subjects were nonsmokers and had no prior history of cardiovascular disease (i.e., hypertension, diabetes, or hyperlipidemia) or orthopedic disease. After receiving a detailed explanation of the study, including the procedures, possible risks, and benefits of participation, each subject signed an informed consent form.

### 2.2. Control of Diet and Physical Activity

Throughout the study, including the washout period (*please refer to the experimental procedures*), the subjects were asked to avoid alcohol consumption and were only allowed to drink water. The diets consumed by the subjects on the night before the day of the study and the breakfast on the day of the study (when resistance exercise was performed in normoxia or hypoxia) were controlled. Briefly, the same dinner (containing: energy 967 kcal, protein 27 g, fat 30.5 g, carbohydrate 146 g, and sodium 1457 mg) and breakfast (energy 320 kcal, protein 3 g, fat 0 g, carbohydrate 80 g, and sodium 112 mg) were prepared for all the subjects. Strenuous exercise was prohibited 48 h before each main study session.

### 2.3. Experimental Procedures

**Figure 1:**
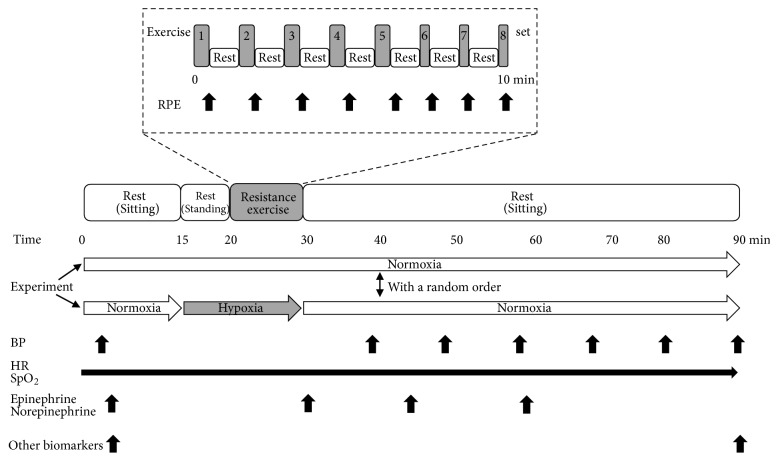
Experimental protocol of the present study. BP: blood pressure, HR: heart rate, and SpO_2_: arterial oxygen saturation. RPE: rate of perceived exertion. Black arrows indicate measuring points and periods.

The subjects were requested to abstain from caffeinated beverages for 12 h before each session and from strenuous exercise and alcohol for 24 h before each session. Throughout all the study sessions, we carefully controlled the room temperature at 20 ± 1°C to prevent the effects of change in BP, and external stimuli were minimized. All subjects performed two trials: (a) normobaric normoxic exercise (room air; equivalent altitude to 400 m) and (b) normobaric hypoxic exercise (fraction of inspired oxygen: FiO_2_ = 0.13). These two trials were performed in a random order and at the same time (8:30 -11:30) of the day to avoid the effect of the circadian rhythm, with at least a 1-week washout period. During this washout period, the subjects were asked to maintain their usual life and to avoid strenuous exercise. In the hypoxic trial, a 5-minute normoxic sitting rest condition was set while breathing room air. After this period, a 10-minute sitting and a 5-minute standing rest, followed by the bilateral leg squat exercise, was set while breathing hypoxic gas (FiO_2_ = 0.13). Thereafter, a 60-minute sitting recovery was set while breathing room air (Figure 1). Hypoxic gas was supplied via a 200 liter Douglas bag reservoir through a two-way, nonrebreathing valve and face mask using a hypoxic generator system (Hypoxico Everest Summit II: Will Co., Ltd., Tokyo, Japan), as previously reported in our recent study [22]. The inspired oxygen concentration was verified before and after each experiment (AE-310; Minato Medical Science, Osaka, Japan). In the normoxic trial, the same protocol as the hypoxic trial was adopted, but the subjects breathed room air throughout the study. The resistance exercise consisted of bilateral leg squats (1 repetition/2 sec) at 50% of 1-RM for eight sets with 1-minute intervals based on a previous study [21] and preliminary test in our laboratory. During the first five sets, they performed 14 reps, while during the last three sets, they performed seven reps. Thus, there was a total of 91 reps for the leg squats (14 reps × 5 sets plus 7 reps × 3 sets). The 1-RM trials were designed using increments of 10 kg until 60–80% of the perceived maximum was reached. The load was then gradually increased by small weights (2.5–5 kg) until lift fail, which was defined as the participant's failure to maintain proper form or to completely lift the weight. Proper technique and a complete range of motion were required for each successful 1-RM trial [23]. For further confirmation, each subject repeated the 1-RM trails, and we confirmed the same 1-RM for all the subjects. No injuries were observed during the 1-RM testing. This test was performed 1 week before the main study. The last acceptable lift with the highest possible load was determined as 1-RM.

### 2.4. Measurements

BP was measured by the oscillometric method (HEM-907, OMRON, Tokyo, Japan) at baseline (pre-exercise) and at 10, 20, 30, 40, 50, and 60 min after exercise. At rest, BP was measured twice, and the average BP of the two measurements was considered as the subject's BP value. We also confirmed that the difference in the SBP or DBP was < 5mmHg compared to the values of one before measurement at rest [24]. According to the technical details provided by the manufacture (Omron, Co., Ltd., Tokyo, Japan), this device can adjust for different arm circumference (e.g., using different width of cuffs). Indeed, the validity of this device (e.g., the values of repeated measurements were different by < 5 mmHg) has been confirmed in previous studies [25–27]. During 60 min recovery period, BP was measured once to minimize the time course effect. Heart rate (HR) and arterial oxygen saturation (SpO_2_) were continuously monitored using a wireless HR monitor (RS 800CX, Polar Electro, Japan) and automatic vital sensor (TM-256, A&D, Tokyo, Japan), respectively, throughout the experiment.

### 2.5. Blood Sampling and Analysis

Venous blood samples (20 ml) were taken from the antecubital vein at baseline and after 60 min of recovery. Samples were immediately centrifuged at 3000 rpm for 15 min at 4°C (MX-300 Tomy Seiko Co, Ltd., Tokyo, Japan) to separate the plasma and serum and were then frozen at -80°C for further analysis of the antidiuretic hormone (ADH), aldosterone, human atrial natriuretic peptide (hANP), adenosine, osmotic pressure (OSM), and free fatty acid (FFA) by SRL Co. Ltd. (Tokyo, Japan).

### 2.6. Data Analysis

The mean arterial pressure (MAP) was calculated using the following:

(1) MAP=systolic  BP  SBP−diastolic  BP  DBP3+DBP

The values of HR and SpO_2_ were adopted at baseline, immediately after each set of leg squats and after 1, 2, 5, 10, 20, 30, 40, 50, and 60 min of recovery for further analysis. These values were averaged during the last 1 min at sitting baseline and during the 10 s immediately after each exercise and each recovery period.

### 2.7. Statistics

The values are expressed as the mean ± SD. Two-way repeated-measures with pairwise (Dunnet) post hoc tests were used to evaluate the changes in all physiological variables across different oxygen levels. First, as these variables were continuous variables, we analyzed them as parametric dataset. However, the sample size was small; therefore, equal variance failed in the variables, and Friedman nonparametric and pairwise (Scheffe) post hoc tests were used. The Spearman correlation coefficient was used for the relationship between changes in the MAP and HR from baseline to the values during recovery periods. A* P* value of less than 0.05 was considered statistically significant. Statistical analyses were performed using commercial software packages (Sigma Stat 3.5; Hulinks, Chicago, IL, USA).

## 3. Results

**Figure 2:**
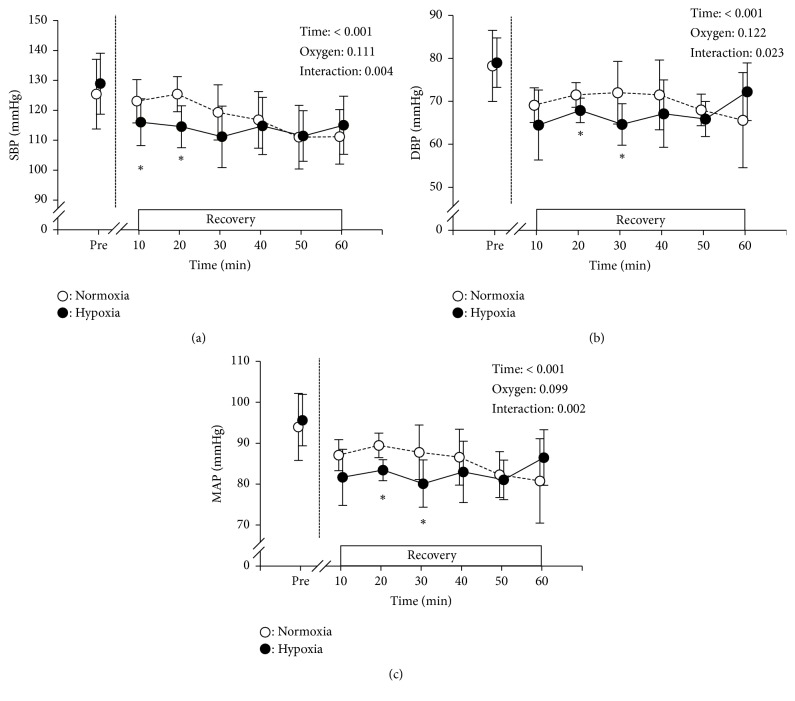
Changes in systolic blood pressure (SBP: panel (a)), diastolic blood pressure (DBP: panel (b)), and mean arterial blood pressure (MAP: panel (c)) at baseline (pre) and during 60 min recovery under normoxia (open circles) and hypoxia (closed circles). *∗* indicates a significant difference between normoxia and hypoxia within the same time period.

The changes in BP variables are shown in Figure 2. There were no significant differences in the pre-exercise values of SBP, DBP, and MAP between normoxia and hypoxia. Significant main effect for time and interaction (time × oxygen) was observed for all BP variables. The SBP after 10 and 20 min of recovery in hypoxia was significantly lower than in normoxia (*P* < 0.05). Similarly, the DBP and MAP after 20 and 30 min of recovery in hypoxia were also lower than in normoxia (*P* < 0.05). Moreover, the SBP after 30 min and the MAP after 10 min of recovery in hypoxia showed lower values than in normoxia (*P *< 0.1).

**Figure 3:**
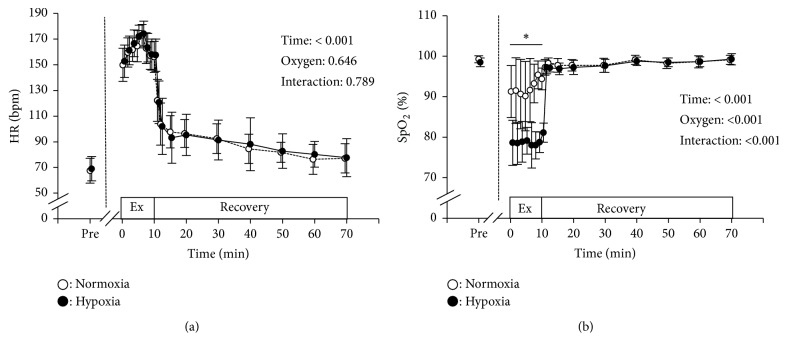
Changes in HR (panel (a)) and SpO_2_ (panel (b)) at baseline (pre) and during exercise and recovery. Circles indicate the same as in Figure 2. *∗* indicates a significant difference between normoxia and hypoxia within the same time period.

The HR during the first five sets of exercise increased from about 150 bpm to 175 bpm, while they decreased slightly during the last three sets to ~160 bpm. After eight sets of exercise, the HR decreased acutely within the first 2 min and then decreased gradually until 60 min after exercise. There were no significant differences in HR at any of the time points between normoxia and hypoxia (Figure 3(a)). SpO_2_ decreased ~10% during exercise in the normoxic trial and ~20% in the hypoxic trial. During recovery, the SpO_2_ in both trials recovered to baseline values within 1 min. There were significant differences in SpO_2_ between trials for each exercise set, but no differences were observed at baseline or during recovery (Figure 3(b)).

Table 1 shows the biomarker changes between normoxia and hypoxia. A significant main effect for time (pre vs. post) was observed in ADH, aldosterone, hANP, and OSM (*P* < 0.05). Additionally, a significant main effect for the condition (normoxia vs. hypoxia) was observed in ADH, resulting in the values for ADH after exercise in hypoxia being significantly higher than in normoxia.

**Figure 4:**
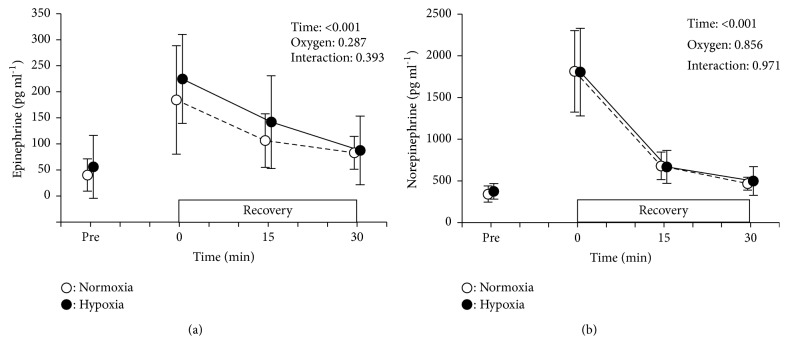
Changes in plasma epinephrine (panel (a)) and norepinephrine (panel (b)) at baseline (pre), immediately after resistance exercise (time = 0) and during the first 30 min of recovery. Circles indicate the same as in Figure 2.

The baseline values (pre-exercise) for both epinephrine and norepinephrine showed similar values between conditions. Immediately after exercise, both values remarkably increased fourfold over the pre-exercise values; thereafter, both parameters decreased in accordance with time changes. No significant differences were observed between trials at any point (Figure 4). Summarized results of the statistical analysis [i.e., F values, effect size (partial *η*^2^) and statistical power (1-*β*)] of the biomarkers and BP variables are shown in Table 2.

**Figure 5:**
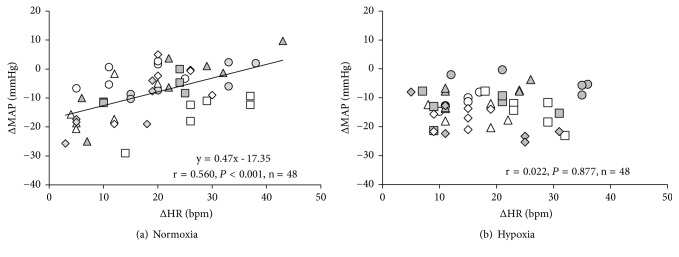
Relationship between changes in MAP and HR under normoxia (panel (a)) and hypoxia (panel (b)) when all data points during the recovery period are pooled. The changes in MAP and HR were calculated between the baseline values and each recovery period (10, 20, 30, 40, 50, and 60 min). Different symbols indicate each subject.

Changes in the MAP from the baseline values during recovery were related to the changes in HR from the baseline values during recovery in normoxia when all data were included (6 points during recovery × 8 participants = 48 plots, Figure 5(a)); however, this correlation was not statistically significant in hypoxia (Figure 5(b)).

## 4. Discussion

To the best of our knowledge, this is the first study to investigate the influence of resistance exercise under hypoxia on postexercise hemodynamics. The major findings of the present study were threefold: (1) the reductions in BP and the increases in ADH responses during recovery after resistance exercise were greater in hypoxia than in normoxia, (2) the HR responses throughout the study were similar between normoxia and hypoxia, and (3) the changes in HR from the baseline values during the recovery period were associated with the MAP changes in normoxia, but this relationship was not observed in hypoxia. Collectively, these results suggest that hypoxic resistance exercise caused greater hypotension after exercise and increased in ADH responses. From observing the results of the relation between HR and MAP during recovery periods, the underlying mechanisms of PEH after resistance exercise may be different between normoxia and hypoxia.

Since MAP is a functional product of cardiac output (HR × stroke volume [SV]) and systemic vascular resistance (SVR), it may at first be reasonable to focus on these two basic components (three parameters). Previous studies have demonstrated controversial findings regarding how these parameters (i.e., cardiac output [CO], SV, and SVR) change after resistance exercise. Some studies found that CO decreased [6, 7, 9, 10], probably due to reductions in SV [7, 9, 10], while one study showed unchanged CO [8]. On the other hand, it was reported that SVR increased [6], remained unchanged [8, 10], or decreased [7] after resistance exercise. Unfortunately, these previous studies were conducted in normoxia and not hypoxia [6–10]; in the present study, we assessed only HR and did not find any differences between trials. Therefore, it is uncertain whether reductions in CO (including SV) and/or SVR caused greater hypotension in hypoxia. However, a previous study reported that there were no differences in HR, SV, and CO between normoxia and hypoxia after exercise [28]. Additionally, it has been reported that endothelial mediated vasodilation was greater in hypoxia than in normoxia after exercise [29]. Although we did not measure blood flow, meaning without parameters of SVR (i.e., MAP/blood flow), there may be a possibility that the greater postexercise hypotension in hypoxia in the present study might be induced by reductions in SVR. It may be worth mentioning that the relationship between the changes in MAP and HR differed between trials despite similar HR responses* per se* in both trials. All the previously mentioned studies above found increases in HR during recovery after resistance exercise, which suggests that this increased HR during the hypotensive period may play an important role in maintaining BP, namely, the prevention of syncope after exercise. A positive relation between these parameters, which was observed in the normoxia trial, may indicate that reductions in MAP are compensated for by a higher HR, while other mechanisms should be considered in the hypoxia trial. Our analysis included all data (i.e., all the subjects and time points); however, interindividual and intraindividual variations should be considered in future studies.

The hypoxia trial led to greater ADH secretion than the normoxia trial. A classic hemodynamics study reported that ADH increased to about two times the pre-exercise level after treadmill walking [30]. Furthermore, it has been reported that acute hypoxic exposure may produce significant hypotension with consequent increased plasma arginine vasopressin (i.e., increased ADH) [31]. Therefore, our results may be partly supported by these studies. In contrast, a subsequent study reported that release of arginine vasopressin was unaffected by hypoxia* per se* (i.e., at resting condition); moreover, arginine vasopressin increased during exercise, and its release was primarily stimulated by increased OSM [32]. Although the study settings of previous studies and the present study were completely different, the results of OSM in the preset study that showed significant decreases from the prevalues in both normoxia and hypoxia seemed inconsistent. We must acknowledge that measured points were only before exercise and 60 min after exercise and, therefore, further explanation about the relationship among changes in MAP, ADH, and OSM during this longer period (more than 60 min) cannot be obtained at this stage. Another concern is the longer exposure in hypoxia. In the present study, the subjects were exposed to hypoxia for ~ 90 min (13% FiO_2_). Similar long exposure to hypoxia ~60 min (10.5% FiO_2_) was present in a previous study [31], while a relatively shorter period of exposure to hypoxia with exercise ~20 min (13% FiO_2_) was reported in another study [32]. Therefore, our results indicated that longer exposure to hypoxia could be a secondary effect of hypoxia, including greater release of ADH and greater reductions in BP after resistance exercise that was in the previous study [31]. Nonetheless, we did not find any significant association or causal relationship between changes in the BP and ADH; therefore, future studies that explore this subject are warranted.

With regard to other biomarkers, we obtained confusing findings. Previous hemodynamic studies highlighted the potential role of exaggerated circulating epinephrine levels in syncope under hypoxia [33, 34]. Indeed, fourfold higher epinephrine levels were found at the onset of hypotension in hypoxia [35]. Moreover, exaggerated circulating epinephrine levels may have been linked with skeletal muscle vasodilation and/or vasodilation or splanchnic circulation [36, 37]. In the present study, the epinephrine and norepinephrine levels immediately after exercise were fourfold higher than the baseline values, but there were no statistical differences between normoxia and hypoxia.

One difficulty in interpreting our results against the previous studies is that we did not assess orthostatic tolerance and no subjects claimed (pre) syncope symptoms throughout the study. By contrast, hANP and adenosine, which are potent vasodilators, decreased or remained unchanged and there were no differences between the trials. These may also be difficulties in interpreting the BP responses during recovery in the present study. However, as the circulating half-life of ANP is only 2-3 min, it is questionable whether hANP release may relate to BP responses [38]. In addition, a previous study reported that decreased BP after exercise was not directly related to the release of ANP [39]. It was also reported that adenosine is responsible for vasodilation during the initial phase after ischemic exercise in dogs, but the re-uptake of adenosine is very rapid following exercise [40]. It is well known that the renin–angiotensin–aldosterone system is a hormone system that regulates BP and fluid balance. Briefly, renin is released from the kidneys during low perfusion pressure; thereafter, it converts angiotensin to angiotensin I, followed by angiotensin II, which is a powerful vasoconstrictor. Angiotensin II also stimulates the secretion of the hormone aldosterone [41]. We found significant increases in aldosterone after 60 min of recovery, but there were no differences in the aldosterone levels between the trials. A classic hemodynamic study found that aldosterone levels increased ~twofold at 15 min after exercise [30]. Therefore, although this is highly speculative with regard to the hypotension period, decreased hANP and increased aldosterone levels may prevent further reductions in BP (i.e., the prevention of syncope).

However, these biomarker results (i.e., epinephrine, norepinephrine, hANP, adenosine, and aldosterone responses) may not account for the different BP regulation between normoxia and hypoxia; therefore, further results are required. Since higher levels of FFA in rats, following acute infusion into the portal vein, mediate the pressor response [42], there may be a possibility that the FFA levels affected the BP response during recovery in the present study. Indeed, a previous study demonstrated that both the FFA and the BP variables increased in parallel after a heparin infusion activated lipoprotein lipase activity and facilitated the turnover of triglycerides to FFAs [43]. In the present study, we found that oxygen, time, and, interaction had no significant effect on the FFA levels; however, the FFA in both trials increased slightly and showed a lower trend in hypoxia 60 min post-recovery. Therefore, our results for BP can be partly explained by previous studies. We must acknowledge that this is also speculative, and we should assesse glucose and lipid metabolism directly in future studies.

### 4.1. Methodological Considerations

There are several limitations in interpreting the results of the present study. First, the relatively small sample size should be considered. Thus, based on our previous study [24], we also conducted post hoc power analysis for pairwise comparisons that were observed with significant differences in variables (i.e., SBP, DBP, MAP, and ADH) as the standard of 80% power with a two-sided significance level of 0.05 (G Power 3.1). We estimated that a sample size of eight would have been necessary to achieve the appropriate statistical power for BP comparisons (SBP at 10 min and 20 min and DBP and MAP at 20 min and 30 min), while 13 subjects would have been required for ADH values postexercise. However, 7 of the 8 subjects showed higher ADH values in hypoxia than in normoxia, and the statistical power was high enough for the BP variables. Although future studies are required, it is unlikely that additional data will strongly affect our conclusions. Second, we recruited only young males in this study. It is therefore uncertain whether our results can be generalized to other populations, such as females and aged people. Similarly, the lack of differences in the physical fitness level of the study subjects should be considered to account for individual differences in future studies. Finally, the precise mechanisms causing greater hypotension after hypoxic resistance exercise than after normoxic resistance exercise are still unclear. We cannot account for this, and therefore the assessment of other potential candidates for influencing BP regulation (e.g., cardiac output, vascular resistance, baroreflex sensitivity, and various vasodilator substances [e.g., nitric oxide, prostaglandins, ATP, and histamine]), alterations in muscle metabolism, and the promotion of angiogenesis may be required in future studies [2, 44].

## 5. Conclusion

Our results demonstrate that bilateral leg squats in hypoxia elicit greater hypotension during recovery than in normoxia. Moreover, ADH release was greater during the recovery period in hypoxia than in normoxia. Changes in HR from baseline during recovery from baseline were related to changes in MAP only in normoxia. These results suggest that the higher HR values during recovery may compensate for hypotension only in normoxia; thus, the underlying mechanisms for the attenuation of hypotension after resistance exercise may differ between normoxia and hypoxia. These findings could help explain the increased risk of syncope after resistance exercise at high-altitudes.

## Figures and Tables

**Table 1:** Blood biomarkers between pre- and poststudy in two different oxygen conditions.

		Normoxia	Hypoxia	Time	Oxygen	Interaction
ADH, pg ml^−1^	Pre	1.48 ± 1.56	1.59 ± 1.16	0.015	0.023	0.220
	Post	2.35 ± 1.39^†^	3.44 ± 2.27^*∗*†^			
Aldosterone, pg ml^−1^	Pre	153 ± 38	161 ± 26	<0.001	0.401	0.336
	Post	267 ± 83^†^	313 ± 87^†^			
hANP, pg ml^−1^	Pre	16.5 ± 7.6	16.1 ± 7.4	0.024	0.966	0.456
	Post	11.8 ± 3.1^†^	12.5 ± 4.6^†^			
Adenosine, u l^−1^	Pre	20.6 ± 8.7	19.3 ± 7.0	0.844	0.350	0.429
	Post	20.9 ± 8.2	19.2 ± 6.1			
OSM, mOsm kgH20^−1^	Pre	283 ± 1	285 ± 2	0.007	0.361	0.402
	Post	280 ± 2^†^	280 ± 4^†^			
Free fatty acid, *μ*Eq L^−1^	Pre	100.4 ± 52.8	94.8 ± 74.0	0.104	0.071	0.204
	Post	207.4 ± 151.5	126.1 ± 61.9			

Values are mean ± standard deviation. ADH: antidiuretic hormone (vasopressin); hANP: human atrial natriuretic peptide.

OSM: osmotic pressure. ^*∗*^*P* < 0.05 between normoxia and hypoxia within the post.

^†^ *P* < 0.05 between pre and post within the same condition.

**Table 2:** Summarized results of statistical analysis in the subset of outcomes.

	F value				Effect size (partial *η*^2^)			Power (1-*β*)		
	*df*	Time	Oxygen	Interaction	Time	Oxygen	Interaction	Time	Oxygen	Interaction
ADH	(1, 7)	8.408	10.365	1.815	0.546	0.597	0.206	0.702	0.788	0.215
Aldosterone	(1, 7)	44.125	0.800	1.066	0.863	0.103	0.132	0.999	0.122	0.146
hANP	(1, 7)	8.262	0.002	0.622	0.541	0.000	0.082	0.695	0.050	0.105
Adenosine	(1, 7)	0.042	1.001	0.704	0.006	0.125	0.091	0.054	0.140	0.113
OSM	(1, 7)	14.044	0.955	0.798	0.667	0.120	0.102	0.893	0.136	0.121
Free fatty acid	(1, 7)	3.495	4.542	1.966	0.333	0.394	0.219	0.366	0.452	0.229
Epinephrine	(3, 21)	18.527	1.327	1.045	0.726	0.159	0.130	1.000	0.170	0.243
Norepinephrine	(3, 21)	92.469	0.035	0.078	0.930	0.005	0.011	1.000	0.053	0.062
SBP	(6, 42)	9.464	3.316	3.761	0.575	0.321	0.350	1.000	0.350	0.933
DBP	(6, 42)	6.451	3.088	2.768	0.480	0.306	0.283	0.997	0.330	0.824
MAP	(6, 42)	8.661	3.629	4.370	0.553	0.341	0.384	1.000	0.377	0.966

*df*: degree of freedom.

## Data Availability

The data used to support the findings of this study are available from the corresponding author upon request.
